# Clozapine prescribing: comparison of clozapine dosage and plasma levels between White British and Bangladeshi patients

**DOI:** 10.1192/bjb.2020.59

**Published:** 2021-02

**Authors:** Rahul Bhattacharya, Leah White, Laura Pisaneschi

**Affiliations:** 1 Tower Hamlets Community Services, East London NHS Foundation Trust; and Barts and the London School of Medicine, UK; 2 East London NHS Foundation Trust, UK; 3 Tower Hamlets Clozapine Clinic, East London NHS Foundation Trust, UK

**Keywords:** Clozapine, ethnopharmacology, clozapine dose, ethnicity

## Abstract

**Aims and method:**

To compare differences in clozapine doses and plasma levels between Bangladeshi and White British patients. Following ethical approval we identified all current Bangladeshi and White British patients on clozapine maintenance in an east London clinic. We carried out univariate and multivariate regression analyses to examine associations between clozapine doses and ethnicity, age, gender, smoking status and weight. We also compared plasma clozapine levels of the two groups.

**Results:**

On univariate analysis White British patients had on average 85 mg higher doses than Bangladeshi patients (*P* = 0.004). Older age, male gender and smoking were also associated with higher dose. On multivariate analysis only age and smoking status remained significant. A greater proportion of Bangladeshi patients had high plasma clozapine levels compared with White British (30.76% *v*. 20.75%), although the difference was not statistically significant.

**Clinical implications:**

Our findings point to the need for the broadening of data collection on ethnic differences in clozapine prescribing within big data-sets such as Prescribing Observatory for Mental Health (POM-UK). Ethnopharmacological variations can inform more person-centred guidance on prescribing.

Clozapine is a unique antipsychotic that demonstrates superior efficacy in people with treatment-resistant schizophrenia and is recommended for treatment in such cases.^1^ The response rate in these individuals is around 40%.^2^ Clozapine has been shown in several pharmacoepidemiological studies to show superior long-term efficacy in terms of reducing hospital admissions.^3^ It also surpasses other antipsychotics in reducing self-harm^4^ and overall mortality.^5^

The pharmacological basis for the unique efficacy of clozapine in treatment-resistant schizophrenia is not clearly understood.^6^ Clozapine treatment is associated with a number of adverse effects.^7–9^ Unlike for most other antipsychotics, the decision to stop clozapine is taken more often by prescribers than by patients, and for reasons of adverse effects more than for lack of efficacy.^10^ The Prescribing Observatory for Mental Health (POM-UK) conducts national audits of clozapine use in the UK, gathering detailed information, for example that clozapine prescribing for people with schizophrenia is higher in men than in women.^11^ Although the POM-UK audit collects ethnicity data, it does not compare prescribing practices or variations between ethnicities.

Ethnopharmacology relates to the study of substances used medicinally by different ethnic groups and examines variations in our body's processing of drugs based on ethnicity, including pharmacogenetics. It is known that prescribing rates for clozapine vary, ranging from less than 10 patients per 100 000 people to nearly 180 patients per 100 000 people.^12^ Variations in dosage may be due to variations in prescribing practice and/or the patient's metabolism. At either end of the spectrum patients may be ‘poor metabolisers’ or ‘ultra-rapid metabolisers’. Metabolic variation can be genetic, but it can also be environmental (e.g. due to cigarette smoking). Ethnic variation in metabolism along the cytochrome P450 has been known for a few decades.^13^ Drug clearance is represented by the concentration-to-dose (C/D) ratio in blood plasma under steady-state and trough conditions. A very low C/D ratio indicates an ultra-rapid metaboliser, whereas a very high C/D ratio indicates a poor metaboliser. In the USA, clozapine C/D ratios, measured in (ng/mL)/(mg/day), typically range from 0.6 (male smokers) to 1.2 (female non-smokers). Inhibitors (including fluvoxamine and oral contraceptives) and inflammation can also increase clozapine C/D ratios.^14^

The British National Formulary recommends titrating clozapine to a dose of 300 mg/day if tolerated, which can be further increased up to a maximum dose of 900 mg/day.^15^ Despite varied estimates of response threshold, plasma levels can be useful in optimising treatment. According to the Maudsley Prescribing Guidelines, in those not responding to clozapine, dose can be adjusted to give plasma levels in the range 0.35–0.5 mg/L (a range reflecting a consensus of the above findings). The guidelines also mention that plasma levels may help in decision-making in those who are not tolerating clozapine, for example by a reduction to a dose guided by the plasma level range mentioned above.^16^ It is documented that plasma levels of clozapine may be higher in ‘Asians’.^17^ This was a study from Singapore in which ‘Asian’ patients were noted to have more than twice the effective clozapine concentration-to-dose ratio than ‘Caucasian’ patients. This very high C/D ratio would suggest a higher prevalence of poor metabolisers in this population. We are not aware of any similar studies in South Asians or in the Bangladeshi population.

We looked at two self-assigned ethnicities as accepted by UK census.^18^ The London Borough of Tower Hamlets (an administrative unit within the city) has a unique ethnic composition, being home to the largest Bangladeshi population in England.^19^ The Bangladeshi population accounts for almost one-third of all residents, closely followed by the second largest ethnic group, White British (31%).^20^ This unique ethnic composition provided a particular opportunity for research into differences in prescribing of clozapine and differences in plasma levels documented between these two ethnic groups. We recognise that our study is not equipped to look into actual genetic and epigenetic differences between the two populations. However, comparing distinct subgroups, i.e. White British and Bangladeshi, we minimise the heterogeneity in each group and make the results more relevant to the populations being studied.

The primary aim of this study was to compare prescribed doses of clozapine in the Bangladeshi population with those in the White British population. The secondary aim was to see whether the odds of plasma clozapine levels being above 0.5 mg/L (the upper limit of the recommended therapeutic range) were increased by belonging to Bangladeshi ethnicity compared with White British.

## Method

All patients receiving clozapine (through the National Health Service) in Tower Hamlets are registered to the Tower Hamlets Clozapine Clinic. All such patients receive clozapine blood monitoring at the clinic and they are registered with the Zaponex Treatment Access System (ZTAS), which is an on-line patient data-base for monitoring treatment.

The study proposal was approved by the ethics committee of the local healthcare provider (East London NHS Foundation Trust). We collected cross-sectional data from the Tower Hamlets Clozapine Clinic as on 21 November 2018.

We defined ‘maintenance dose’ as a dose for which the patient is no longer having blood monitoring as frequently as weekly, which generally indicates that the patient has been taking clozapine for at least 18 weeks after dose titration. We took this as a proxy measure for maintenance dose of clozapine for the purpose of the study. On 21 November we identified 79 Bangladeshi and 52 White British patients receiving clozapine in their maintenance phase. We carried out univariate linear regression analyses for ethnicity, age, gender, smoking status and weight, followed by multivariate regression analyses on the variables that were statistically significant on univariate analysis.

For our secondary aim we searched for results of clozapine plasma level tests conducted on the participants. Of the results available in the clinic's records, 78 were obtained from the people of Bangladeshi ethnicity and 53 were from the people of White British ethnicity. We calculated how many of these results were above 0.5 mg/L. Subsequently we analysed whether the odds of an individual recording a plasma level above 0.5 mg/L was increased if they were of Bangladeshi ethnicity compared with White British ethnicity.

The study did not gather any new data but analysed data that were already available in the clinic's electronic patient records.

## Results

Ethnicity, age, gender and smoking status were all significantly associated with clozapine dose (significance was borderline for gender) (Table 1). White British participants received on average 85 mg/day higher doses of clozapine than Bangladeshi participants. Older people received on average 39 mg/day higher doses for each decade of increasing age. Women received on average 67 mg/day lower doses than men, but the difference was only borderline significant. Non-smokers received on average 81 mg/day lower doses than smokers. Weight was not significantly associated with dose in our sample.

**Table 1:** Clozapine dose analysis

			Univariate analysis		Multivariate analysis		
	Mean dose, mg/day	Mean dose difference, mg/day	95% CI	*P*	Difference	95% CI	*P*
Dose by ethnicity							
White British (*n* = 52)	435.1	85.4	(27.5 to 143.3)	0.004*	51.3	(−12.3 to 114.8)	0.11
Bangladeshi (*n* = 79)	349.68						
Dose by smoking status							
Smokers (*n* = 59)	427.97	79.38	(137.8 to 23.7)	0.006*	69.9	(129.9 to 10.0)	0.023*
Non-smokers (*n* = 72)	348.59						
Dose by gender							
Female (*n* = 36)	334.72	−67.38	(−131.9 to −2.9)	0.041*	−35.43	(−101.8 to 31.0)	0.29
Male (*n* = 95)	402.1						
Dose by weight							
Weight (per 10 kg)		1.2	(−15.9 to 13.5)	0.87			
Dose by age							
Age (per decade)		38.9	(12.3 to 65.6)	0.005*	29.6	(0.3 to 58.8)	0.048*

* *P* < 0.05.

When we tested variables known to influence clozapine dose in a multivariate model comparing age (per decade), gender, smoking status and ethnicity, only age and smoking status remained statistically significant. Difference based on ethnicity was no longer statically significant at the 5% level, although the trend was towards White British having higher doses. As weight was not significantly associated in univariate analysis, we did not carry out multivariate analysis on this variable.

Of the 78 results of plasma clozapine level tests obtained from Bangladeshi participants, 24 (30.76%) had levels higher than the upper limit of the therapeutic range. Of the 53 results obtained from White British participants, 11 (20.75%) had levels higher than therapeutic range. The odds ratio of a higher (above 0.5 mg/L) plasma level in a Bangladeshi individual was calculated to be 1.697, although this was not statistically significant as the confidence interval crossed 1 (Table 2).

**Table 2:** Odds ratio of a plasma clozapine level higher than the upper limit of the recommended therapeutic range by ethnicity

Ethnicity	Plasma level >0.5 mg/L, *n* (%)	OR (95% CI)
Bangladeshi (*n* = 78)	24 (30.76)	1.697 (0.748–3.852)
White British (*n* = 53)	11 (20.75)	

## Discussion

### Key findings

We are aware of several factors that could affect clozapine dose. We found that Bangladeshi participants, women, younger participants and non-smokers received lower doses. When we looked at our dosage data using the multivariate model only, smoking status and age were statistically significant. However, it is possible that we were not sufficiently powered with our sample size to demonstrate the difference in dosing between Bangladeshi and White British ethnicity. There was a trend towards White British participants needing higher doses. We are aware that other factors might also have influenced dosage (e.g. co-prescribing), but this information was not available in the clinic's regular monitoring records.

We examined records of plasma clozapine levels to explore whether higher plasma clozapine concentrations were more likely to be reported in those of Bangladeshi ethnicity compared to White British ethnicity. We found that, despite receiving lower doses, Bangladeshi patients were more likely to have higher plasma concentrations of clozapine when tested. This would support the suggestion of a higher prevalence of high C/D ratios and poor metaboliser status among Bangladeshi patients taking clozapine. For simplicity of analysis, we used the Maudsley Prescribing Guidelines reference therapeutic plasma level to analyse the plasma level data as a binary variable. This study is not designed to assess the therapeutic plasma range for clozapine. The data-base for clozapine plasma levels did not have a record of associated variables. We used existing clinic data for our analysis and were limited by the data that were routinely collected.

### Bangladesh and Bangladeshi populations

Bangladesh is a new country, created in 1971 from a division of Pakistan decided on the basis of linguistic differences (eastern Pakistan was predominantly Bengali-speaking), and previously separated from British India on the basis of religion when Colonial rule ended in 1947. Bangladesh is in the eastern part of South Asia, which has a high internal ethnic homogeneity, with 98% identifying themselves as ‘Bangalees’.^21^ In fact some residents of Tower Hamlets arrived in the UK before the country was formed and many are second generation. It is also recognised that people from the Sylhet region are the strongest subgroup within this population. However, they also identify themselves as Bangladeshi. Ethnicity is a different construct in each society and may merge with local notions of ‘race’, national identity or other invented traditions.^22^

There is a body of literature in cultural psychiatry in which the UK's Bangladeshi population has been studied (e.g. in Mental Health Act detention data). Information such as ours has implications in terms of global health and can inform prescribing in other countries, especially in Bangladesh, a country of 169 million people. Schizophrenia is the most common diagnosis in mental health settings in Bangladesh, according to a World Health Organization report across in-patient units, mental hospitals and community-based clinics.^23^ We believe that such information and guidance has significant public health implications both in Bangladesh, as well as for migrant populations of Bangladeshi origin across the globe. We also believe that assuming large populations are monolithic, for example as implied by concepts such as ‘Asian’ and ‘Caucasian’, risks overgeneralisation and misses out on differences within these groups. Having smaller clearer groups might allow a granularity in our understanding that would otherwise not be possible.

### Ethnicity, pharmacology and study populations

Ethnicity is reported to be an important, but often ignored factor in psychopharmacology. A number of ethnically specific variations have been found in the genetic and non-genetic mechanisms affecting pharmacokinetics and dynamics of psychotropic drugs, which might underlie differences in drug prescribing and response across ethnicities. Although some of these ethnic differences might be partially explained by genetic factors, a number of ethnically based variables such as diet and cultural attitudes could potentially have a significant impact.^24^ This might include differences in smoking habits between Bangladeshi and White British patients or levels of comorbidity. Very few studies have analysed biological basis and metabolic variations in relation to clozapine. A notable exception is the above-mentioned 2005 study from Singapore and even then there are difficulties with what the terms Asian and Caucasian mean.^17^ We acknowledge that our study design does not offer the opportunity to explore these variables in detail. Although there has been some research into ethnic variation in clozapine tolerability and effective dosing, a significant evidence base is still lacking.

Most studies in the field are case–control studies such as ours, comparing small samples of broad ethnic entities or case series, sometimes with a more distinct ethnic group. The 2005 Singapore study comparing 20 ‘Asian’ patients from Singapore with 20 ‘Caucasian’ patients from Australia reported that the mean clozapine dose for the Asian group was 176 mg/day, whereas for the ‘Caucasian’ group it was 433 mg/day.^17^ A more recent study found that ‘East Asians’ (Chinese in the sample) had a clinically relevant reduced clozapine clearance (suggesting higher prevalence of poor metabolisers) compared with ‘Caucasians’ (Italians in the sample).^25^ However, the ethnic groups ‘Asian’, ‘East Asian’ and ‘Caucasian’ are, in our opinion, too broad and heterogeneous to safely generalise the findings in a clinical setting.

We also discovered that findings were not always consistent. Results from a study conducted in south London by the South London and Maudsley NHS Trust reported no significant differences in clozapine dosage prescriptions between in-patients from White, Black and Asian ethnic groups.^26^ Although the overall study sample was large, the clozapine sample for which ethnicity was noted was only 188 and included only in-patients, whereas we compared all patients on clozapine (community and in-patients). As the south London study also included all ethnicities, once again we would argue the categories were too broad. The 11 ‘Asian’ patients included in the study did receive a lower mean dose of clozapine but this was not statistically significant. In another recent study the researchers concluded that clozapine bioavailability did not vary between Maori and European patients.^27^ Therefore one needs a more nuanced approach rather than generalising diverse minority groups as monolithic.

Studies that examined more coherent ethnic identities lacked control groups. A review of 1256 records from Novartis Pakistan (one of the monitoring systems for clozapine treatment) were analysed and the average maintenance dose was found to be 230 mg/day.^28^ A study involving 162 Taiwanese patients with refractory schizophrenia reported a mean dose of 379.5 mg/day (range: 100–900 mg/day).^29^ The only other study on the Bangladeshi population was a small case series comprising 21 patients in a tertiary care centre in Bangladesh, which revealed that most of the patients with treatment-resistant schizophrenia (64%) responded to clozapine doses of 50–200 mg/day and the remaining patients who responded to treatment required doses of 250–500 mg/day.^30^ In these reports without a control group one can argue that prescriber factors such as prescribing culture, habits or even cost could have influenced the prescribed dose as opposed to patient factors.

### Clinical and research implications

We believe that the information obtained from our study is important as it provides an opportunity to explore variation in tolerability and effective dosage controlled for prescriber factors. Even with relatively small numbers we found a statistically significant difference in dosing of clozapine. Although we did not find statistically significant odds of high plasma levels in Bangladeshi participants it is possible that the study was not sufficiently powered to elicit the statistical significance. Of note, high plasma levels were reported in Bangladeshi participants despite the lower mean prescribed dose, indicating a higher C/D ratio and possibly higher prevalence of poor metabolisers in the Bangladeshi population.

For more comprehensive exploration of these issues, we believe there is need to analyse ‘big data’. POM-UK audits have the opportunity to do this. We would like this national audit to analyse dosage and tolerability variation data across ethnicity. Similarly, data on plasma clozapine levels held in central repositories might offer sufficiently large samples to enable study of ethnic variations and could steer research in cytochrome-P450 variations between populations. If such variation is clearly documented, it could inform prescribing guidelines on a more cautious and conservative approach when titrating patients of Bangladeshi ethnicity on clozapine.

We also suggest that studies of ethnic variations in clozapine doses and plasma levels should select more coherent ethnic groups and be mindful of heterogeneity within minority populations.

## Data Availability

The data that support the findings of this study are available from the corresponding author, R.B., upon reasonable request

## About the authors

**Rahul Bhattacharya** is a Consultant Psychiatrist and Associate Clinical Director for Community Services in Tower Hamlets, East London NHS Foundation Trust and an Honorary Senior Clinical Lecturer at Barts and the London School of Medicine, UK. **Leah White** is a Consultant Old Age Psychiatrist with East London NHS Foundation Trust and an Associate Fellow of the Higher Education Academy, London, UK. **Laura Pisaneschi** is Lead Nurse for the Tower Hamlets Clozapine Clinic, East London NHS Foundation Trust, UK.

## Supplementary material

For supplementary material accompanying this paper visit https://doi.org/10.1192/bjb.2020.59. click here to view supplementary material

## References

[1] Keating D, McWilliams S, Schneider I, Hynes C, Cousins G, Strawbridge J, Pharmacological guidelines for schizophrenia: a systematic review and comparison of recommendations for the first episode. BMJ Open 2017; 7: e013881. 10.1136/bmjopen-2016-013881 PMC5223704 28062471

[2] Siskind D, Siskind V, Kisely S. Clozapine response rates among people with treatment-resistant schizophrenia: data from a systematic review and meta-analysis. Can J Psychiatry 2017; 62: 772–7. 28655284 10.1177/0706743717718167 PMC5697625

[3] Kesserwani J, Kadra G, Downs J, Shetty H, MacCabe JH, Taylor D, Risk of readmission in patients with schizophrenia and schizoaffective disorder newly prescribed clozapine. J Psychopharmacol 2019; 33: 449–458. 30616489 10.1177/0269881118817387 PMC6431783

[4] Wimberley T, MacCabe JH, Laursen TM, Sørensen HJ, Astrup A, Horsdal HT, Mortality and self-harm in association with clozapine in treatment-resistant schizophrenia. Am J Psychiatry 2017; 174: 990–8. 28750580 10.1176/appi.ajp.2017.16091097

[5] Cho J, Hayes RD, Jewell A, Kadra G, Shetty H, MacCabe JH, Clozapine and all-cause mortality in treatment-resistant schizophrenia: a historical cohort study. Acta Psychiatr Scand 2019; 39: 237–47. 10.1111/acps.12989 PMC6492259 30478891

[6] Khokhar JY, Henricks AM, Sullivan EDK, Green AI. Unique effects of clozapine: a pharmacological perspective. Adv Pharmacol 2018; 82: 137–62. 29413518 10.1016/bs.apha.2017.09.009 PMC7197512

[7] Nielsen J, Young C, Ifteni P, Kishimoto T, Xiang YT, Schulte PF, Worldwide differences in regulations of clozapine use. CNS Drugs 2016; 30: 149–61. 26884144 10.1007/s40263-016-0311-1

[8] Segev A, Evans A, Hodsoll J, Whiskey E, Sheriff RS, Shergill S, Hyoscine for clozapine-induced hypersalivation: a double-blind, randomized, placebo-controlled cross-over trial. Int Clin Psychopharmacol 2019; 34: 101–7. 30614850 10.1097/YIC.0000000000000251

[9] Shirazi A, Stubbs B, Gomez L, Moore S, Gaughran F, Flanagan RJ, Prevalence and predictors of clozapine-associated constipation: a systematic review and meta-analysis. Int J Mol Sci 2016; 17: 863. 10.3390/ijms17060863 PMC4926397 27271593

[10] Legge SE, Hamshere M, Hayes RD, Downs J, O'Donovan MC, Owen MJ, Reasons for discontinuing clozapine: a cohort study of patients commencing treatment. Schizophr Res 2016; 174: 113–9. 27211516 10.1016/j.schres.2016.05.002 PMC5756540

[11] Prescribing Observatory for Mental Health. Topic 18a: The Use of Clozapine. POMH-UK, 2019.

[12] Taylor D, Werneke U. Ethnopharmacology. Nord J Psychiatry 2019; 72(supp 1): S30–2.

[13] Aitchison KJ, Jordan BD, Sharma T. The relevance of ethnic influences on pharmacogenetics to the treatment of psychosis. Drug Metabol Drug Interact 2000; 16: 15–38. 10820581 10.1515/dmdi.2000.16.1.15

[14] de Leon J. Personalizing dosing of risperidone, paliperidone and clozapine using therapeutic drug monitoring and pharmacogenetics. Neuropharmacology 2020; 168: 107656. 31150659 10.1016/j.neuropharm.2019.05.033

[15] Joint Formulary Committee. *British National Formulary* (BNF) 78: September 2019 – March 2020. BMJ Publishing Group/Royal Pharmaceutical Society, 2019.

[16] Taylor D, Barnes TRE, Young AH. The Maudsley Prescribing Guidelines in Psychiatry (13th edn). Wiley Blackwell, 2018.

[17] Ng CH, Chong SA, Lambert T, Fan A, Hackett LP, Mahendran R, An inter-ethnic comparison study of clozapine dosage, clinical response and plasma levels. Int Clin Psychopharmacol 2005; 20: 163–8. 15812267 10.1097/00004850-200505000-00007

[18] Cabinet Office. List of ethnic groups. GOV.UK, 2020 (https://www.ethnicity-facts-figures.service.gov.uk/ethnic-groups).

[19] Tower Hamlets Council. Ethnicity in Tower Hamlets: Analysis of 2011 Census Data (Research Briefing 2013-01). GOV.UK, 2013 (https://www.towerhamlets.gov.uk/Documents/Borough_statistics/Ward_profiles/Census-2011/RB-Census2011-Ethnicity-2013-01.pdf).

[20] Tower Hamlets Council. Ethnic Profile: White Other Population in Tower Hamlets. GOV.UK, 2015 (https://www.towerhamlets.gov.uk/Documents/Borough_statistics/Census_2011/Ethnic_profile_White_Other_20_06_15.pdf).

[21] Murshid TM. State, nation, identity: the quest for legitimacy in Bangladesh. In The Post-Colonial States of South Asia: Political and Constitutional Problems (eds A Shastri, A Jeyaratnam Wilson): 165. Curzon Press, 2001.

[22] Hobsbawm EJ, Ranger T (eds). The Invention of Tradition. Cambridge University Press, 1983.

[23] World Health Organization, Ministry of Health & Family Welfare Bangladesh. WHO-AIMS Report on Mental Health System in Bangladesh. WHO, 2007.

[24] Chaudhry I, Neelam K, Duddu V, Husain N. Ethnicity and psychopharmacology. J Psychopharmacol 2008; 22: 673–80. 18308818 10.1177/0269881107082105

[25] Ruan C-J, Zang YN, Wang CY, Cheng YH, Sun C, Spina E, Clozapine metabolism in East Asians and Caucasians: a pilot exploration of the prevalence of poor metabolizers and a systematic review. J Clin Psychopharmacol 2019; 39: 135–44. 30811372 10.1097/JCP.0000000000001018

[26] Taylor D. Prescribing of clozapine and olanzapine: dosage, polypharmacy and patient ethnicity. Psychiatr Bull 2004; 28: 241–3.

[27] Menkes DB. Steady-state clozapine and norclozapine pharmacokinetics in Maori and European patients. EBioMedicine 2018; 27: 134–7. 29254680 10.1016/j.ebiom.2017.11.030 PMC5828556

[28] ur Rahman R, Siddiqi MN, Ansari M, Saeed F. The under use of clozapine in Pakistan (Oral free paper). JPAN, 2014; 3: 52.

[29] Chang WH, Lin SK, Lane HY, Hu WH, Jann MW, Lin HN. Clozapine dosages and plasma drug concentrations. J Formos Med Assoc 1997; 96: 599–605. 9290269

[30] Uddin MS, Ahmed S, Yasir SM. Profile of clozapine therapy: a cross sectional piloting in a tertiary care setting of Bangladesh paper. J Psychiatry Psychiatric Disord 2017; 1: 190–8.

