# Supplementary material for: Clozapine prescribing: comparison of clozapine dosage and plasma levels between White British and Bangladeshi patients
Source: BJPsych Bull. 2021 Feb;45(1):22–7. doi: 10.1192/bjb.2020.59 (PMC8058897; doi:10.1192/bjb.2020.59)
Supplement: Supplementary file 1 [file S2056469420000595sup.zip › S2056469420000595sup003.pdf]

ICMJE INTERNATIONAL COMMITTEE of MEDICAL JOURNAL EDITORS

ICMJE Form for Disclosure of Potential Conflicts of Interest

Section 1. Identifying Information

1. Given Name (First Name) LAURA
2. Surname (Last Name) PISANESCHI
3. Date 8/11/19

4. Are you the corresponding author? ☐ Yes ☑ No

5. Manuscript Title
A COMPARISON OF EFFICACY AND TOLERABILITY OF CLOZAPINE IN PATIENTS OF BANGLADESHI AND WHITE BRITISH ETHNICITY

6. Manuscript Identifying Number (if you know it)
~~[illegible]~~
UNKNOWN

Section 2. The Work Under Consideration for Publication

Did you or your institution **at any time** receive payment or services from a third party (government, commercial, private foundation, etc.) for any aspect of the submitted work (including but not limited to grants, data monitoring board, study design, manuscript preparation, statistical analysis, etc.)?

Are there any relevant conflicts of interest? ☐ Yes ☑ No

Section 3. Relevant financial activities outside the submitted work.

Place a check in the appropriate boxes in the table to indicate whether you have financial relationships (regardless of amount of compensation) with entities as described in the instructions. Use one line for each entity; add as many lines as you need by clicking the "Add +" box. You should report relationships that were **present during the 36 months prior to publication**.

Are there any relevant conflicts of interest? ☐ Yes ☑ No

Section 4. Intellectual Property -- Patents & Copyrights

Do you have any patents, whether planned, pending or issued, broadly relevant to the work? ☐ Yes ☑ No

ICMJE INTERNATIONAL COMMITTEE *of* MEDICAL JOURNAL EDITORS

ICMJE Form for Disclosure of Potential Conflicts of Interest

Section 5. Relationships not covered above

Are there other relationships or activities that readers could perceive to have influenced, or that give the appearance of potentially influencing, what you wrote in the submitted work?

☐ Yes, the following relationships/conditions/circumstances are present (explain below):

☑ No other relationships/conditions/circumstances that present a potential conflict of interest

At the time of manuscript acceptance, journals will ask authors to confirm and, if necessary, update their disclosure statements. On occasion, journals may ask authors to disclose further information about reported relationships.

Section 6. Disclosure Statement

Based on the above disclosures, this form will automatically generate a disclosure statement, which will appear in the box below.

NOTHING TO DECLARE

Evaluation and Feedback

Please visit http://www.icmje.org/cgi-bin/feedback to provide feedback on your experience with completing this form.
